# On Chronic Organic Liver Affections

**Published:** 1855-10

**Authors:** A. W. Griggs

**Affiliations:** Newnan, Ga.


Article III.—*On Chronic Organic Liver Affections.* By A. W. Griggs, M. D., of Newnan, Ga.

This climate seems to be peculiarly adapted to the generation of the various affections of the liver common to the greater part of the South; indeed, it is very difficult to find an individual who has never experienced the unpleasant effects of liver disease. Three-fourths of the diseases which here elicit the attention of the physician either arise from, or are complicated with, derangement of the hepatic secretory function. Nearly every cause which militates against the harmonious actions of the different organs of the human system exerts some influence over the operations of the biliary apparatus. Hence we conclude that no medical subject is more entitled to mature reflection and consideration than the nature and treatment of those serious maladies usually denominated Liver Complaints. Our object in writing this article is to demonstrate that the different varieties of chronic organic affection of the liver require very different treatment. We are aware that Calomel and Blue Pill, in any disease of the liver whatever, are the first remedies thought of by most of the old school physicians in our country. Truly, many of them would despair of relieving any case without these "time honored" preparations. Now, we wish that they—the remedies—were able to meet all the indications presented in these very common diseases. But we have noticed the fact that the universal administration of mercurials, in these cases, is not only inadequate to a perfect cure, but often inadmissible and contra-indicated on account of the precise nature of the disease, and also the constitutional peculiarities of the patient. We meet with complications so often that we are convinced of the improbability of certainty, it matters not what system of practice be adopted. We in vain look for a specific for these affections, which every quack says he can cure. Science acknowledges her utter destitution of specifics in these affections, and puts to the blush all the pretended discoveries that are swelled in such contemptible characters in newspapers, hand-bills, and almanacs, to decoy the public mind into error, and to enhance the selfish interests of the proprietor. Yet we are happy to know that the scientific medical practitioner, by modifying his treatment to suit the different pathological conditions of the organ, is frequently able to eradicate the dangerous developments of the most hopeless case. We believe, however, that with the generality of us we are accustomed to go ahead and prescribe too indiscriminately for every variety of the disease under consideration. We mean no offence, for the best, or in other words, most successful practitioner of medicine, deprived of rest by day and of sleep by night, will not arouse his intellect to proper scrutiny in examining a chronic affection of so great frequency, and one, too, which does not immediately threaten the dissolution of his patient. As a distinguished physician once remarked "he has become so much of a workman that he forgets to be a philosopher." We have often asked physicians what is the matter with Mr. A., and the response would be he has "Liver Complaint." This answer was never satisfactory to our mind. We always considered it too indefinite to convey any just idea of the true character of the disease; for we are convinced of the necessity of discrimination, so far as to separate the different pathological states of the liver into distinct classifications, which individually require, at least, some modifications of the general treatment. We beg leave to illustrate the position by the report of two or three interesting cases which we have been called upon to treat.

CASE 1.—Mrs. ——, wife of a respectable farmer of Troup County, Georgia, æt. 38 or '9, had been the unhappy victim of disease many years. Happening at the residence of her husband, about twelve months ago, we were requested to see the case and prescribe whatever we might consider necessary.

*Examination.*—We found her to be a very delicate lady, much addicted to a melancholy state of mind; of a sallow complexion and evidently of the bilious temperament; she suffered with symptoms of indigestion; there was a continual uneasy sensation in the right hypochondrium; urine tinged with the coloring matter of bile; subject to constipation of the bowels; fecal discharges were colorless; had no cough; the whites of her eyes exhibited a yellow cast. The inferior edge of the liver could not be detected beneath the cartilages of the ribs.

*Physical Signs.*—Upon percussion, the sounds appeared to emanate from a solid and elastic body beneath the parieties of the right hypochondriac region. No other peculiarity was detected.

*Previous Condition of the Patient.*—She informed me that she was a stout, healthy girl, and continued so until she was married and became the mother of several children, and that her health began to give way about ten or twelve years ago; that she was subject to bilious attacks; also to severe paroxysms of headache; that several years since she had a serious attack of acute Hepatitis.

*Diagnosis.*—We were of the opinion that the acute Hepatitis terminated or resulted in contraction and induration of the substance of the liver.

*Treatment.*—We recommended alterative doses of Pil. Hydrarg, to be taken every other day and night until slight ptyalism should be produced. Then to suspend the remedy for a week and to resume it again and continue it until the same effect should be experienced. Then to suspend two weeks and again resume and continue as before, and so on to persevere until the liver should be aroused to a healthy action and assume its natural condition in respect to size, etc. In connection with this, we ordered the patient to be sponged over the region of the liver with dilute Nitro Muriatic Acid, and as soon as these means could be discontinued, we recommended, as a tonic, a bitter made of Peruvian bark and precip. Carb. Ferri, placed in Madeira wine. The treatment was persevered in for several months, and she regained her wonted health.

Of chronic organic disease of the liver we believe that cases like this are the sort which require mercurial medication. We know that mercury acts like a charm wherever it is indicated, but we doubt whether it is so often indicated as used. We see that it acts well in many cases of chronic Hepatitis in which there is no enlargement of the organ, and in which the fibrin of the blood can be diminished without impoverishing the system of the subject; but where there is no enlargement, we think that an opposite course should be adopted, at least so far as the general treatment is concerned. The preparations of Potassa and Iodine are the most reliable remedies in the treatment of chronic glandular enlargements. We have had good success in treating enlarged liver with these preparations, and will weary your patience only with the report of one case:

CASE 2.—On the 8th of June last we visited Mrs. ——, wife of a respectable merchant of Newnan, Georgia, in the 25th year of her age. She had been sick for weeks.

*Examination.*—We found her laboring under the awful apprehension that she was most probably the subject of incurable pulmonary affection. She was reclining supinely on a bed, with her body bent from left to right, so as to increase as much as possible the intercostal spaces on the right side of the thorax. She preferred this position. The right shoulder was drawn up and the left down. She informed me that when first attacked she could only rest on the right side, but that lately she could lie with more ease in the position just described. Her features were shrunken and pallid, with occasional flushings of the cheeks. The mucus membrane of the throat and mouth was nearly colorless. Coughing was incessant, and respiration difficult. There was head ache, cold extremities, and pain in the right side ; there was pain in the right shoulder, extending down the arm ; she was quite gloomy and depressed in feelings ; had become considerably emaciated ; her voice was weak ; talking always excited violent and painful paroxysms of coughing ; her skin had a yellow hue and she suffered with constipation.

*Physical Signs.*—Percussion over the left lung gave no evidences of disease. The sounds emitted from the superior part of the right lung were a little abnormal, and there was some dullness of sound from the inferior portion of the middle lobe. The right hypochondriac region emitted dull, flat sounds, especially from its superior half.

*Diagnosis.*—This was evidently a case of enlargement of the right lobe of the liver. We think it needless to assign a reason for this conclusion, as the stated symptoms will convince the unprejudiced mind of any well-informed and intelligent physician.

*Treatment.*—Ten grains of the Iodide of Potassum were given in the compound syrup of Sarsaparilla twice per diem. Tartar Emetic Ointment was rubbed over the right hypochondrium to produce slight pustulation. No cough mixture was prescribed, as we believed cough due to a mechanical obstruction of the inferior portion of the lung, produced by the tumid liver below. The bronchi may also have been in a state of sympathetic irritation. Vegetable diet was forbidden. The meat of wild fowls and of squirrels was allowed, and in a fortnight she was able to walk over her room, apparently well of her disease, but very weak and very much emaciated.

We now recommended the syrup of the Iodide of Iron, in twenty drop doses, three times per diem, and moderate exercise in an open carriage once a day. She rapidly recovered, and is in fine health at this date.

We have treated one other case, like case second, complicated with uterine hemorrhage, but time will not now permit us to report it. We have only contrasted two cases in order to demonstrate that there are some hepatic derangements that are curable without the use of mercury—the sheet anchor of the old fogies' hopes.

